# Selective extraction of lithium from acidic chloride leachates of spent batteries

**DOI:** 10.1038/s41598-026-43332-y

**Published:** 2026-03-25

**Authors:** Usman Saleem, Vanja Buvik, Sulalit Bandyopadhyay, Hanna K. Knuutila

**Affiliations:** 1 Department of Chemical Engineering, Norwegian University of Science and Technology, 7941 Trondheim, Norway; 2 Department of Chemical Engineering, Particle Engineering Centre, Norwegian University of Science and Technology, 7941 Trondheim, Norway; 3 SINTEF Industry, 7465 Trondheim, Norway

**Keywords:** Lithium, Direct lithium extraction (DLE), Solvent extraction, Battery recycling, Water stripping, Hydrometallurgy, Black mass, Chemistry, Environmental sciences, Materials science

## Abstract

Solvent extraction of lithium (Li) from acidic leachates of spent batteries black mass (BM) over nickel (Ni), manganese (Mn), and cobalt (Co) can allow easy integration of Li recycling into conventional hydrometallurgical flowsheets, eliminating the need for thermal pretreatment of BM. In this study, a ternary organic phase consisting of iron (Fe^3+^), tributyl phosphate (TBP), and 2-ethylhexyl phosphonic acid mono 2-ethylhexyl ester (P507) to selectively extract Li^+^ over Ni^2+^, Mn^2+^, and Co^2+^ has been developed. The extraction, scrubbing, stripping, and regeneration conditions were optimized, and McCabe–Thiele diagrams were constructed. An increasing P507/Fe (optimum 1.5–1.7) mole ratio was found to enhance Li release during water stripping but suppressed extraction. FTIR spectroscopy confirmed the stability of Fe^3+^ in the organic phase during water stripping. The Fe^3+^ preloaded solvent gave > 90% Li (7.7 g/L initially) extraction at an organic/aqueous phase ratio of 7 in four stages. After six cycles, the Li extraction efficiency was maintained, showing excellent selectivity, reducing NMC metal ions from 7.6–64 g/L to only 0–0.05 g/L in purified strip solution. The developed solvent system facilitates water stripping, eliminating the need for acids and alkalis, and allows easy integration in established chloride-based battery recycling processes, enabling early Li recovery from the leachate.

## Introduction

Lithium (Li) is a critical element finding application in Lithium-ion batteries (LIBs), which are playing a central role in decarbonizing the energy sector as an efficient energy storage device. The demand for LIBs has skyrocketed due to rapid adoption in electric vehicles (EVs) and stationary energy storage over the past few years, consequently increasing the demand for Li. To meet this demand, it is imperative to secure a secondary supply of Li from recycling end-of-life batteries. However, LIBs are a heterogeneous material consisting of elements like Li, nickel (Ni), cobalt (Co), manganese (Mn), aluminum (Al), copper (Cu), iron (Fe), graphite, etc., making preferential extraction of Li difficult^1,2^.

There are two main processes to recycle LIBs; pyrometallurgy and hydrometallurgy, and an emerging process, called direct recycling, which directly regenerates the cathode material^2,3^. Pyrometallurgy is a high temperature process where LIBs are treated at high temperature (> 1000 °C) and Li can be recovered in either slag or flue dust, which needs extensive processing^2,4^. Hydrometallurgy utilizes wet chemistry to first leach the metals in solution using an acid (e.g., hydrochloric acid), followed by stepwise recovery of different metals either by solvent extraction or precipitation^2,3^. In both these routes, Li is often recovered as the last element, which is often uneconomical due to Li losses in previous steps, accumulation of impurities (due to pH adjustment, precipitation reagents, etc.), and diluted streams. To solve this challenge, research has been directed towards early-stage Li recovery from the black mass (BM), which is essentially crushed LIB powder^5–8^. In such a process, spent LIBs or BM are thermally treated at ~ 550 °C to decompose the binder coating from the particles and phase transform the Li in the cathode into a water-soluble Li product such as carbonate, chloride, sulphate, etc.^5–8^. Li is then separated by water leaching, and transition metals are left in the filter cake along with graphite^5–8^. This approach is effective in recovering Li; however, it comes at a higher energy cost for thermal treatment, the release of gases that need scrubbing, and adds more process steps.

A similar approach of early Li recovery from the BM can be applied and easily integrated into a conventional hydrometallurgical LIBs recycling process by using a Li selective material. Such material should directly extract Li over Ni, Mn, Co, Fe, Cu, and Al in acidic media after leaching, making it the first element to be recovered from a total leachate. To explore such a material, we extensively reviewed the available Direct Lithium Extraction (DLE) materials and technologies in our previous work^9^. Solvent extraction was found to be the most viable option to perform this extraction in highly acidic conditions, as sorbents (aluminum hydroxide, manganese oxide, titanium oxide, etc.) dissolve and membranes degrade^9^. However, this extraction is often challenging even for solvents because most commercial solvents bind high valence metal ions (Ni^2+^, Co^2+^, Mn^2+^, etc.) over Li in acidic pH, e.g., Cyanex 272, D2EHPA^10^. One way to perform this extraction is by employing tributyl phosphate (TBP) and ferric chloride (FeCl_3_) as the co-extractant. Such a system forms a Li.FeCl_4_.nTBP ion pair complex in the organic phase, given three conditions are satisfied: (1) Cl^−^ concentration of ~ 6 mol/L, (2) Fe^3+^/Li^+^ molar ratio of 1.3–1.7, (3) ≥ 0.01 mol/L hydrochloric acid (HCl) to prevent Fe^3+^ hydrolysis^10,11^. Such extraction systems are extensively studied for Li extraction from brines where Na/Mg.FeCl_4_.nTBP is preloaded in the organic phase from a saturated solution of sodium chloride or magnesium chloride with a quantified amount of FeCl_3_ and HCl, equation (Eq. 1)^10^. Li is then extracted from the brine or leachate by an ion exchange between Na^+^ (or alternatively Mg^2+^) and Li^+^, Eq. (2). Such methodology has been reported to achieve high separation factors of Li/Mg 347 and 435.5 from brines^11,12^. Wesselborg et al.^10^ employed such an extraction system to extract Li from a model acidic leachate of spent batteries, reporting separation factors of Li over Ni and Co of 2825 and 854, respectively.

1 $${\mathrm{Na}}_{{({\mathrm{aq}}.)}}^{ + } + {\mathrm{FeCl}}_{{{\mathrm{4(aq}}.{)}}}^{ - } + {\mathrm{nTBP}}_{{\left( {{\mathrm{org}}.} \right)}} = {\mathrm{Na}}.{\mathrm{FeCl}}_{{4}} .{\mathrm{nTBP}}_{{\left( {{\mathrm{org}}.} \right)}}$$

2 $${\mathrm{Na}}.{\mathrm{FeCl}}_{4} .{\mathrm{nTBP}}_{{\left( {{\mathrm{org}}.} \right)}} + {\mathrm{Li}}_{{\left( {{\mathrm{aq}}.} \right)}}^{ + } = {\text{ Li}}.{\mathrm{FeCl}}_{4} .{\mathrm{nTBP}}_{{\left( {{\mathrm{org}}.} \right)}} + {\text{ Na}}^{ + }_{{\left( {{\mathrm{aq}}.} \right)}}$$

3 $${\mathrm{Li}}.{\mathrm{FeCl}}_{4} .{\mathrm{nTBP}}_{{\left( {{\mathrm{org}}.} \right)}} + {\mathrm{HCl}}_{{\left( {{\mathrm{aq}}.} \right)}} = {\mathrm{H}}.{\mathrm{FeCl}}_{4} .{\mathrm{nTBP}}_{{\left( {{\mathrm{org}}.} \right)}} + {\text{ LiCl}}_{{\left( {{\mathrm{aq}}.} \right)}}$$

4 $${\mathrm{H}}.{\mathrm{FeCl}}_{4} .{\mathrm{nTBP}}_{{\left( {{\mathrm{org}}.} \right)}} + {\mathrm{NaOH}}_{{\left( {{\mathrm{aq}}.} \right)}} = {\mathrm{Na}}.{\mathrm{FeCl}}_{4} .{\mathrm{nTBP}}_{{\left( {{\mathrm{org}}.} \right)}} + {\mathrm{H}}_{2} {\mathrm{O}}_{{\left( {{\mathrm{aq}}.} \right)}}$$

However, the challenge occurs during stripping this complex as it becomes necessary to maintain a high Cl^−^ concentration to prevent Fe^3+^ release, and is usually achieved using HCl either alone or with a salt, e.g., sodium chloride (NaCl), Eq. (3)^11,13^. The stripped organic phase is regenerated or saponified using a base such as sodium hydroxide (NaOH), Eq. (4)^11,13^. For example, the loaded organic phase was stripped with 6 mol/L HCl, achieving 2 mol/L of Li in the strip solution^10^. Another study employed 1 mol/L HCl + 2 mol/L NaCl, achieving 0.474 mol/L Li with 2 mol/L NaCl in the strip solution^14^, and many more^11,12^. Such a high concentration of acids and salts yields a highly acidic and contaminated strip solution, which needs further neutralization and purification steps before Li salt crystallization, in addition to corroding the equipment^13^. To eliminate the use of acids and bases, a cosolvent that complexes with Fe^3+^ can be added, enabling water stripping and solvent regeneration. The Fe^3+^ is speciated back to FeCl_4_^−^ when its forming conditions are met in the aqueous phase (acidity, Cl^−^ concentration, etc.) during the next Li extraction cycle^13,15–17^. Although such methods have been reported for Li extraction from brines, there are no known studies on these solvent systems for Li extraction from industrial BM leachates produced by HCl leaching. The implementation of such a solvent system on battery leachates should meet several prerequisites, for example, avoid dilution and impurity introduction because the raffinate would still contain NMC metals representing the most value, should be able to survive acidic conditions, limit the competition of H^+^ with Li^+^, and the cosolvent should not extract NMC metals over Fe^3+^, to name a few. However, if implemented successfully, it can allow significant savings on acids and alkalis, easy integration in conventional hydrometallurgical flow sheets, and most significantly it can eliminate the need for thermal pretreatment of BM to phase change the Li into water soluble species. Furthermore, DLE studies from brines have reported that the system is recyclable as Fe^3+^ stabilizes in the organic phase, but the effect of continuous cycling on extraction and stripping performance is seldom explored experimentally.

Herein, we present a ternary organic phase consisting of Fe^3+^, TBP, and 2-ethylhexyl phosphonic acid mono 2-ethylhexyl ester (P507) to selectively extract Li^+^ over Ni^2+^, Mn^2+^, and Co^2+^. The extraction, scrubbing, stripping, and regeneration conditions were optimized, and McCabe–Thiele diagrams were constructed. Two process schemes were studied, which differ from each other in how the 1st extraction is performed. The purpose of this was to avoid precipitation during the first extraction. The extraction mechanism was studied with FTIR spectroscopy to confirm the stability of Fe^3+^ in the organic phase during water stripping. The overall Li recovery process (extraction, scrubbing, stripping, and regeneration) was repeated 6 times for scheme 1 and 3 times for scheme 2. The developed approach allows easy integration of Li recycling in conventional hydrometallurgical chloride-based battery recycling processes.

## Materials and methods

### Materials

Hydrochloric acid fuming 37% (HCl, ACS, ISO, Reag. Ph Eur), lithium chloride (LiCl, ACS reagent, ≥ 99%), iron chloride hexahydrate (FeCl_3_.6H_2_O, ACS, Reag. Ph Eur), nickel chloride (NiCl_2_), nickel chloride hexahydrate (NiCl_2_.6H_2_O, ReagentPlus®), oxalic acid (C_2_H_2_O_4_, 98%), tri-n-butyl phosphate (TBP, ≥ 99.0%) were bought from Sigma Aldrich, sodium hydroxide (NaOH, 98.8%, pellets Reag. Ph. Eur.) from VWR chemicals, and potassium sulfate (K_2_SO_4_, 99 + %, ACS reagent) and kerosene (low odor) from Thermo Scientific. The three types of BMs used to produce HCl leachates were provided by an industrial partner wherein the modules were shredded under an inert atmosphere to < 20 mm, dried under reduced pressure at ~ 100 °C to remove volatiles and sieved < 53 µm.

## Analytical methods

The aqueous phase was analyzed by microwave plasma atomic emission spectrometer (MP-AES, 4210 Agilent) to quantify the concentration of metal ions, with relative standard deviation (RSD) of ~ 5% from triplicate measurements. A calibration curve (0–50 mg/L) was made using a multi metal standard solution from Certipur® (1000 mg/L) and the samples were diluted in 2% nitric acid. The concentration in the organic phase was calculated by mass balance. The chloride (Cl^−^) concentration in the aqueous phase was measured by ThermoFisher Scientific Dionex Integrion chromatograph. The Cl^−^ ion standard for IC from Sigma Aldrich (TraceCERT®—10,000 mg/L in water) was used to make a calibration curve (0–40 mg/L) and the samples were diluted accordingly in Milli Q water, with RSD of 2% from triplicate measurements. The free acid concentration in the leachate was measured using a method adapted from^18^. A buffer solution of 50% saturated K_2_SO_4_ and 0.1 M oxalic acid was prepared. The pH of 20 mL buffer solution was adjusted to 6.5–7, using NaOH, and 0.25–0.5 mL of the sample was added and titrated with 0.1 M standard NaOH solution till neutral. The RSD of free acid analysis was within 2%. Fourier-transform infrared (FT-IR) spectroscopy was recorded on a Bruker VERTEX 80 V spectrometer in a range of 4000–400 cm^−1^ with a resolution of 4 cm^−1^ and 50–100 scans to study the complexes formed in organic phase. UV–visible (UV–vis) spectroscopy was done on the organic phase using Agilent Cary 60 UV–Vis spectrometer. The scans were done in the range of 200–800 nm and the samples were prepared in fisherbrand Macro Quartz Cuvettes with a 10.0 mm Path Length.

## Experimental methods

Solvent extraction experiments were performed in a centrifuge tube (15 mL or 50 mL) where aqueous and organic phase were mixed in a shaker (IKA® HS 501 digital) at 280 rpm for 30 min, Fe^3+/^Li^+^ molar ratio of 1.3–1.7^11^, and ambient temperature, unless otherwise mentioned. The phases afterwards were separated either by a separatory funnel or centrifugation (MULTIFUGE™ X1 Centrifuge by Thermo SCIENTIFIC™) at 4000 rpm for 3 min. Solvent extraction experiments involving only TBP were conducted by using organic phase of 80% TBP and 20% kerosene as diluent. This was done to avoid third phase formation^10,11^. The extraction efficiency was calculated by Eq. (5), organic /aqueous (O/A) phase ratio by Eq. (6), distribution ratio (D) by Eq. (7), and separation factor (β) by Eq. (8)^15^.

5 $$\% Extraction = \frac{{C_{f} V_{f} - C_{r} V_{r} }}{{C_{f} V_{f} }}*100$$

6 $$\frac{O}{A} phase\, ratio = \frac{{V_{O} }}{{V_{A} }}$$

7 $$Distribution\, ratio \left( D \right) = \frac{{C_{org} }}{{C_{aq} }}$$

8 $$Separation\, factor \left( \beta \right) = \frac{{D_{Li} }}{{D_{M} }}$$

Where *C*_*f*_ and *C*_*r*_ represent the concentration (g/L), *V*_*f*_ and *V*_*r*_ are the volumes (L) of aqueous feed (before extraction) and aqueous raffinate (after extraction), respectively. *V*_*o*_ and *V*_*a*_ are the volumes (L) of organic and aqueous phase, respectively. *C*_*org*_ and *C*_*aq*_ represent the equilibrium amount (moles) in organic and aqueous phase, respectively. *D*_*Li*_ and *D*_*M*_ is the distribution ratio of Li and metal M, respectively. A select number of experiments were repeated more than 5 times and the repeatability was found to be around 5%.

## Results and discussion

### Characterization of BM leachates

The industrial BMs were leached with 6 mol/L HCl at 80 °C and a solid/liquid (S/L) ratio of 250 g/L for 60 min. The resulting leachates served as the starting feed for subsequent solvent extraction experiments. The leachates were analyzed using MP-AES for quantifying metal ions, IC for chloride, and titration for free acid analysis, and the composition is shown in Table 1. The concentration of transition metal ions is high in all the leachates, especially Ni in NMC 622 and 901. Although Cu, Al, and Fe are recovered during the dismantling and pretreatment stages, some contamination from these metal ions still exists in the leachates^9^. The Cl^−^ concentration in all the leachates is greater than150 g/L, which is due to the high concentration of HCl used during leaching. This is beneficial for the solvent extraction process, as a high Cl^-^ concentration, > 6 mol/L, favors Li extraction^10^. During the leaching reaction, Eq. (8), chlorine gas (Cl_2_) is released as transition metals are reduced to lower oxidation states, which leads to the consumption of Cl^−^ ions^19,20^. The free acid concentrations in all leachates are approximately 1 mol/L, suggesting that either a higher S/L ratio or a slightly less concentrated acid could be employed for the leaching process.

9 $$3{\mathrm{Li}}({\mathrm{NiMnCo}})_{\frac{1}{3}} {\mathrm{O}}_{2(s)} + 12{\mathrm{HCl}}_{{\left( {{\mathrm{aq}}.} \right)}} \to {\mathrm{NiCl}}_{{2\left( {{\mathrm{aq}}.} \right)}} + 3{\mathrm{LiCl}}_{{\left( {{\mathrm{aq}}.} \right)}} + {\text{ MnCl}}_{{2\left( {{\mathrm{aq}}.} \right)}} + {\text{ CoCl}}_{{2\left( {{\mathrm{aq}}.} \right)}} + 6{\mathrm{H}}_{2} {\mathrm{O}} + 1.5{\mathrm{Cl}}_{{2\left( {\mathrm{g}} \right)}}$$

**Table 1:** Composition of industrial BMs leachates used for Li extraction.

BM	Fe (g/L)	Cu (g/L)	Ni (g/L)	Co (g/L)	Li (g/L)	Mn (g/L)	Al (g/L)	Cl- (g/L)	Free acid (mol/L)
NMC 111	1.4 ± 0.08	2.8 ± 0.0	25.2 ± 0.4	22.7 ± 0.1	8.1 ± 0.04	20.0 ± 0.2	0.4 ± 0.02	155.1 ± 0.5	0.7 ± 0.002
NMC 622	1.8 ± 0.04	3.1 ± 0.07	46.4 ± 1.02	14.0 ± 0.4	8.9 ± 0.2	15.9 ± 0.4	2.3 ± 0.02	183.3 ± 0.3	0.8 ± 0.003
NMC 901	3.7 ± 0.02	1.7 ± 0.0	63.6 ± 0.5	7.6 ± 0.1	7.9 ± 0.1	0.0 ± 0.0	0.7 ± 0.0	174.1 ± 1.8	0.9 ± 0.006

### Preliminary direct lithium extraction from BM leachates

**Fig. 1:**
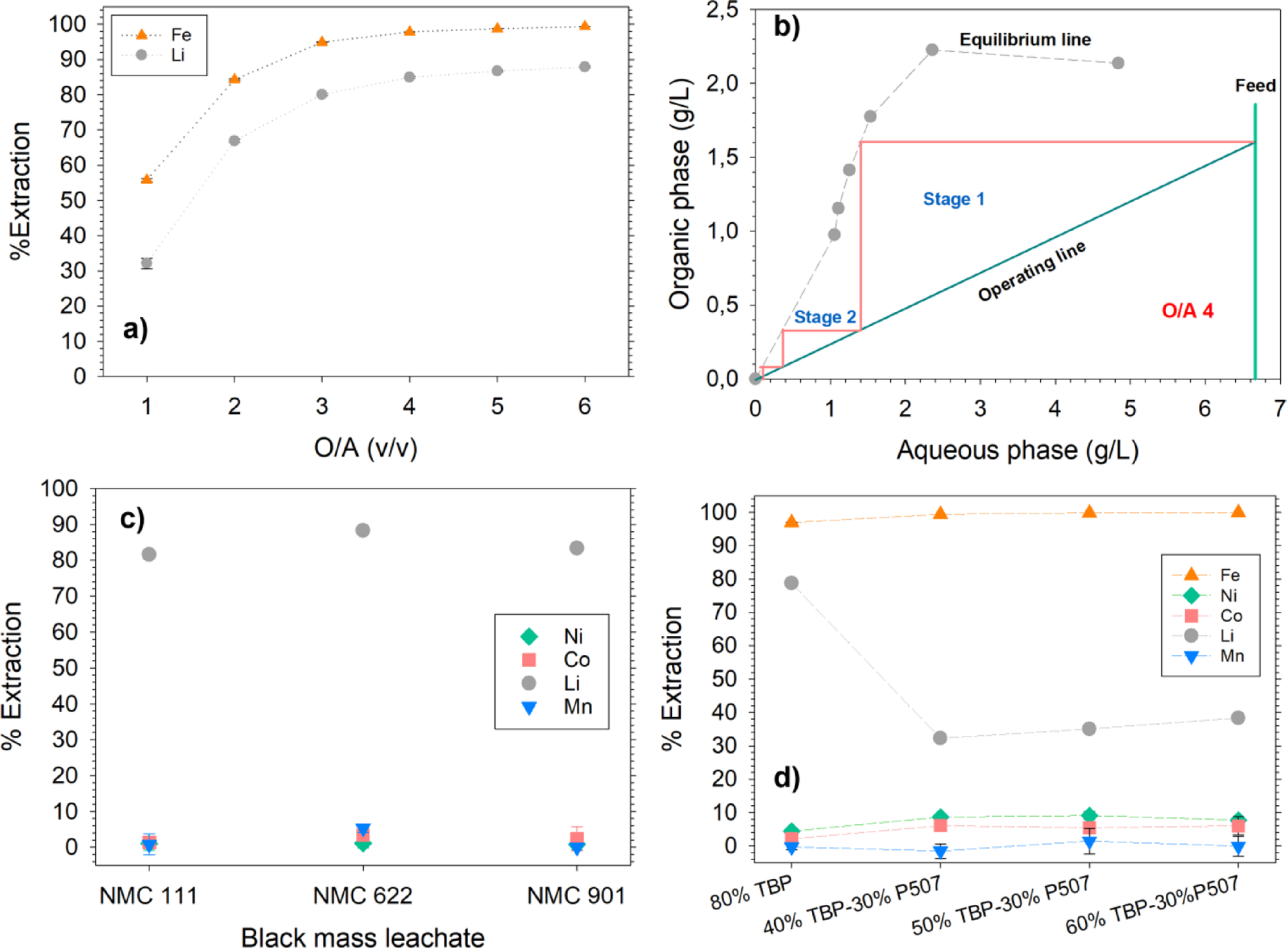
(**a**) Li extraction from 0.5 mol/L NiCl_2_ spiked NMC 622 leachate at different O/A phase ratios using 80% TBP (**b**) McCabe–Thiele diagram for Li extraction by using data from 1a, (**c**) Li extraction from industrial BM leachates conditions.: O/A 4 and spiked with 0.5 mol/L NiCl_2_ except 901 leachate using 80% TBP, (**d**) Li extraction from 0.5 mol/L NiCl_2_ spiked NMC 111 leachate using TBP + P507 in kerosene at O/A 4.

**Fig. 2:**
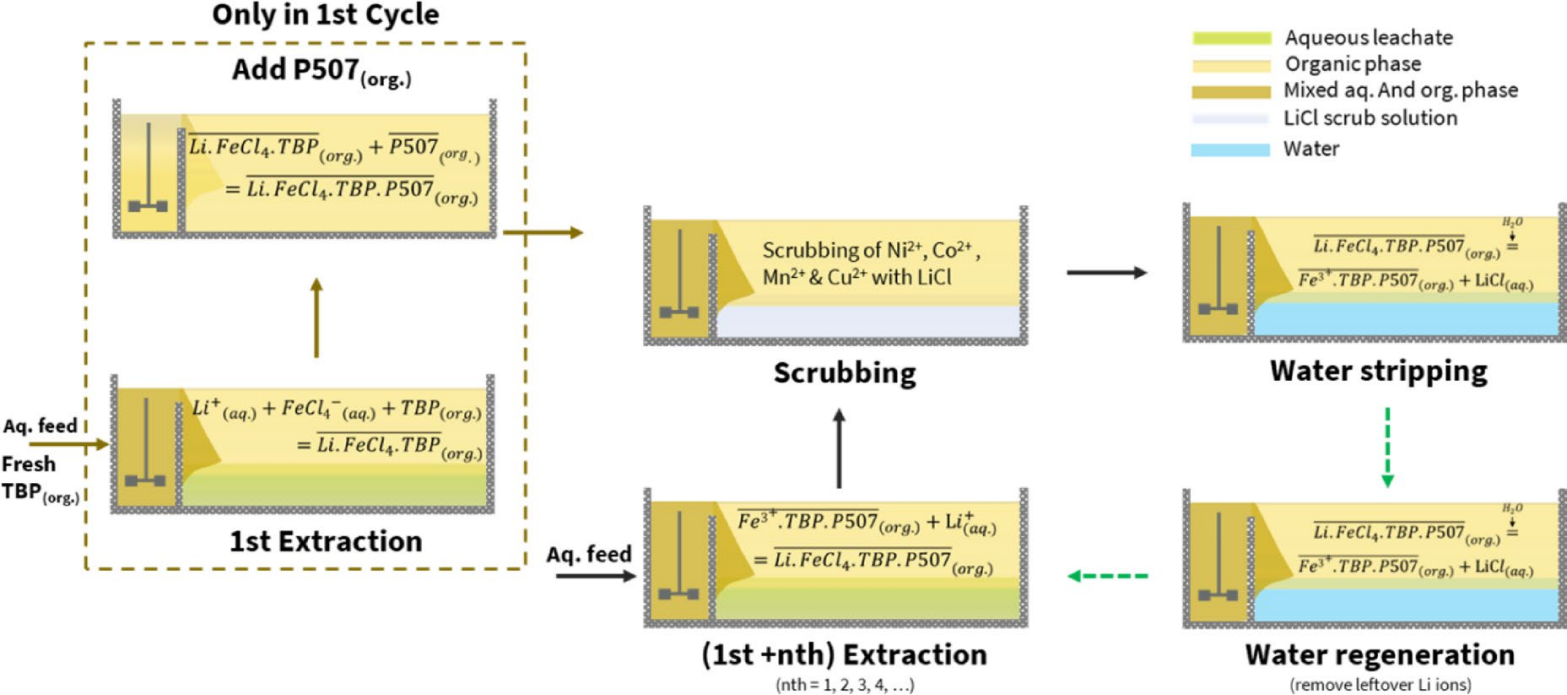
Process scheme 1.

There are two main methods to initiate the extraction process. First, Na^+^ and FeCl_4_^−^ can be loaded in TBP from a stock solution, forming Na.FeCl_4_.nTBP complex in the organic phase. The loaded organic phase can then be used to extract Li^+^ from the BM leachates by an ion exchange^10^. However, as shown in Table 1, the concentration of Li in all the leachates exceeds 1 mol/L. Consequently, 1 mol/L of Na^+^ would be exchanged for Li^+^ during extraction from a preloaded solvent. The ion exchange mechanism would introduce a significant amount of Na^+^ impurity (1 mol/L represents 23 g/L) into the leachate containing Ni, Co, and Mn, which represents the most value. The accumulation of Na^+^ in the leachate poses major challenges as it degrades the purity of NMC products due to coprecipitation and a large amount of sodium salt in the aqueous waste stream that needs further processing^21,22^. To avoid contaminating the leachate, Li.FeCl_4_.nTBP can be directly loaded from the BM leachate by adding FeCl_3_, ensuring that the Fe/Li molar ratio is 1.3–1.7^11^. Therefore, NMC 622 leachate was spiked with FeCl_3_, and solvent extraction was done with only TBP at different O/A phase ratios, Fig. 1a. The Li extraction increased gradually from 30% at O/A 1 and plateaued 80% at O/A 4, after which no appreciable increase was observed. O/A 4 was therefore chosen as an optimum when using TBP alone. The McCabe–Thiele diagram for extraction was constructed, Fig. 1b, and 2 counter-current stages were found to be necessary to extract > 95% Li from a BM leachate containing ~ 7 g/L. The optimized conditions with only using TBP were tested on NMC 111 and NMC 901 leachates as well, and the results are shown in Fig. 1c along with NMC 622. Around 80% Li could be recovered from all the leachates with around 1.2%, 3.4% and 5.3% extraction of Ni, Co, and Mn, respectively. High separation factors were achieved for Li from all the leachates, for example 426, 321, and 541 over Ni, Co, and Mn, respectively, for NMC 111 leachate. High valence metal ions are strongly hydrated compared to monovalent ions, resulting in higher Li extraction^10^. The control experiments were done by using pristine leachates, without adjusting the Fe^3+^/Li^+^ molar ratio, and no Li extraction was observed, while NMC extraction was around 5–10%. Furthermore, an attempt was made to extract Li from FeCl_3_ spiked NMC 111 leachate using different volumes of TBP with 30% P507, Fig. 1d. The different volumes of TBP and P507 were selected based on the methodology adopted by^16^. It was found that Li extraction decreased from 80% (when using TBP alone) to 40%, with Ni, Co and Mn extraction of ~ 10%, across all combinations of TBP and P507. P507 has been reported to suppress Li extraction and has a strong affinity for Fe^3+^ ^16,17^. The order of extraction for P507 has been proposed to be Fe^3+^  > NMC metal ions^23^. The high concentrations of Fe^3+^, necessary to maintain a Fe^3+^/Li^+^ molar ratio of 1.3, likely contributed to the lower Li extraction since both solvents preferentially extract Fe^3+^ over Li.FeCl_4_ due to a substantial concentration gradient. This is also observed in Fig. 1d, where Fe^3+^ extraction is > 99% when P507 is used with TBP. To encourage FeCl_4_^−^ extraction, it is recommended to separate TBP and P507 in the first cycle or reduce the concentration of Fe^3+^ in the leachate. The latter will suppress Li extraction as the Fe^3+^/Li^+^ ratio will be reduced greatly, and that’s why process scheme 1, Fig. 2, was investigated based on the former.

### Process scheme 1

Figure 2 illustrates Process Scheme 1, which was developed to maximize the loading of Li.FeCl_4_ into TBP directly from the BM leachate during the first extraction. An attempt was made to add P507 to the loaded TBP organic phase after 1st extraction, and this was performed only during the first cycle. Following this, the organic phase underwent scrubbing, stripping, regeneration, and the next (1st + nth) extraction. The optimization based on process scheme 1 is detailed in the following sections.

#### Optimization of P507/Fe^3+^ molar ratio, scrubbing and stripping of organic phase

**Fig. 3:**
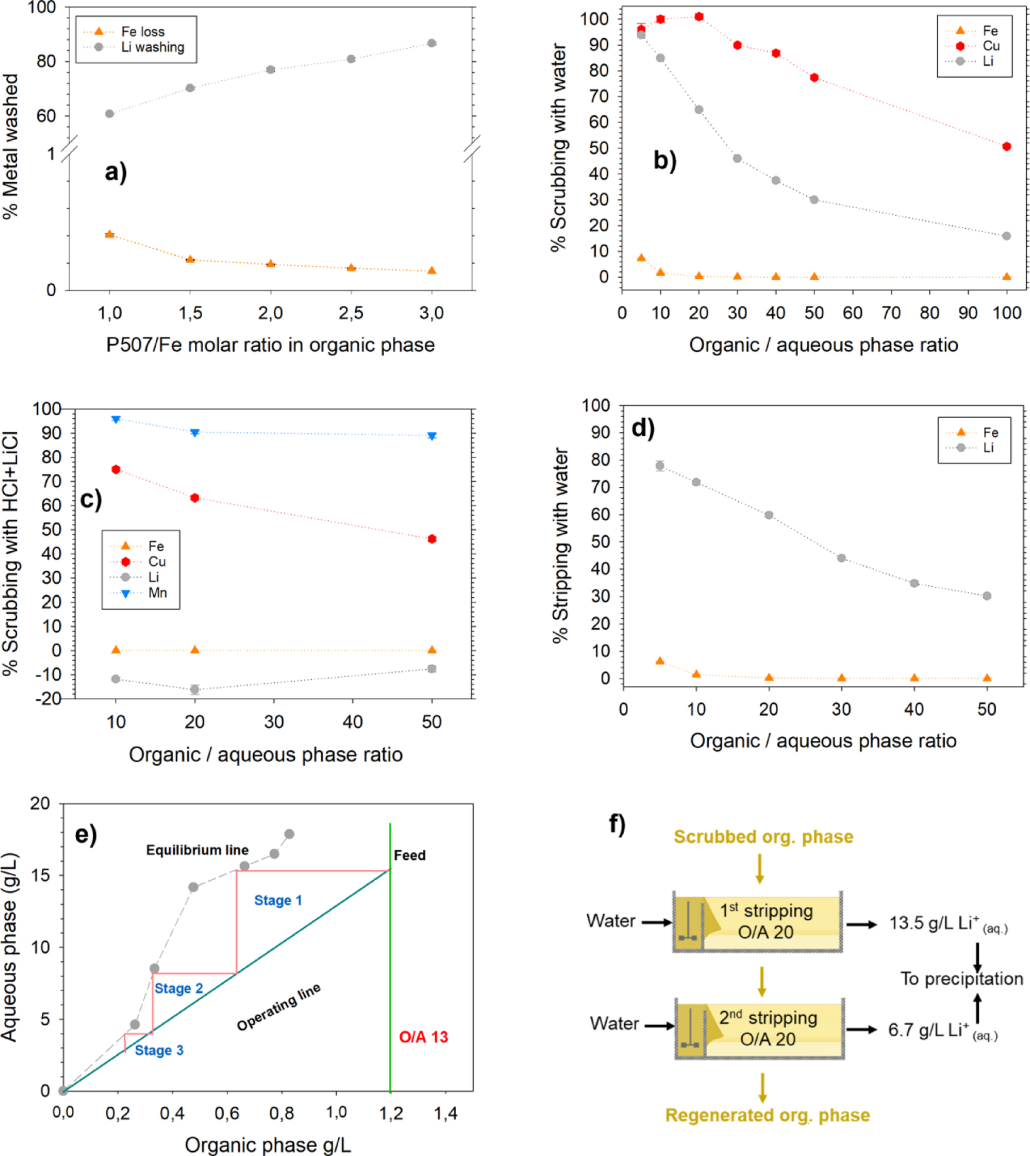
(**a**) P507/Fe^3+^ molar ratio optimization by water washing the loaded organic phase, from 0.5 mol/L NiCl_2_ spiked NMC 111 leachate, at O/A 20, (**b**) Scrubbing of loaded organic phase (P507/Fe^3+^ molar ratio 1.5–1.7) with water, (**c**) Scrubbing of loaded organic phase (P507/Fe^3+^ molar ratio 1.5–1.7) with 0.5 mol/L HCl + 3.5 M mol/L LiCl, (**d**) % Water stripping vs O/A ratio of scrubbed organic phase with 0.5 mol/L HCl + 3.5 mol/L LiCl at O/A 10 , (**e**) McCabe-Theile diagram for counter current water stripping based on 3d, (**f**) Two stage cross-current water stripping at O/A 20 of scrubbed organic phase with 0.5 mol/L HCl + 3.5 mol/L LiCl at O/A 10.

**Fig. 4:**
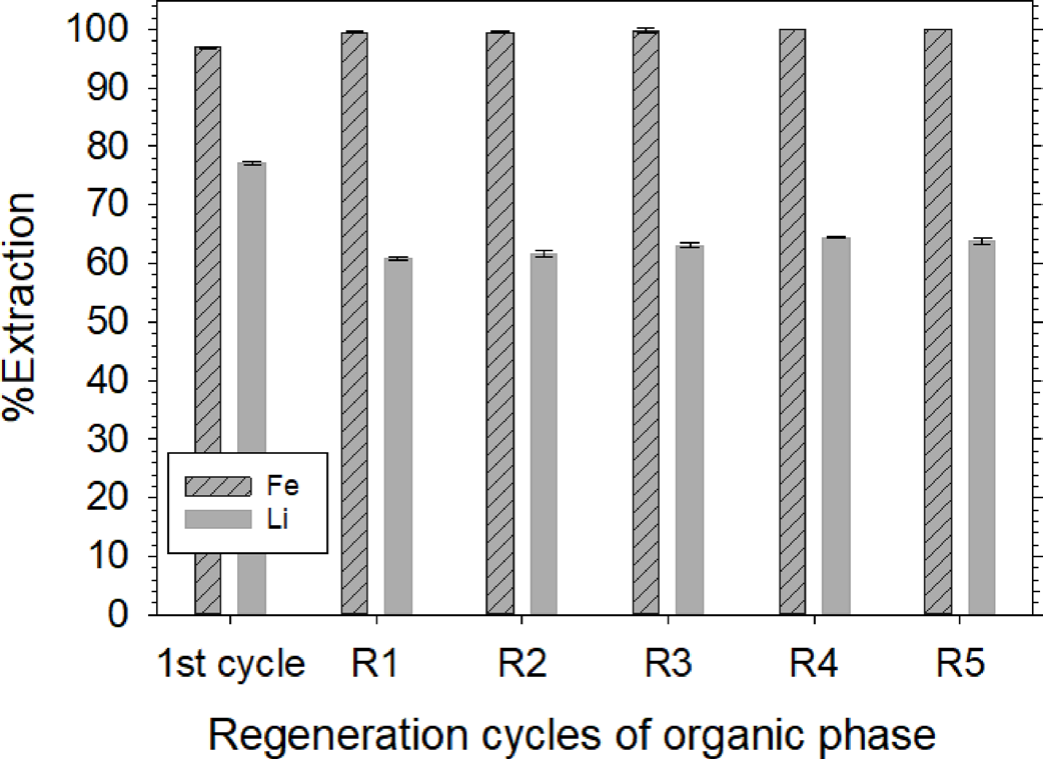
Regeneration cycles of organic phase based on process scheme 1, 1st cycle performed with 0.5 mol/L NiCl_2_ spiked NMC111 leachate with 80%TBP at O/A 4, Cycles R1-R5 performed with 1 mol/L NiCl_2_ spiked NMC901 leachate with Fe^3+^.nTBP.mP507 organic phase at O/A 7.

After adding P507 to the loaded TBP organic phase, it was stripped or washed with water at O/A 20, initially chosen based on the application of a similar system from brines^16^, Fig. 3a. It was found that increasing the P507/Fe^3+^ molar ratio in the organic phase reduced Fe^3+^ loss during water stripping but enhanced Li water washing. Specifically, a higher ratio led to greater Li stripping and lower Fe^3+^ loss, albeit at the cost of reduced Li extraction, also illustrated in Fig. 1d. A ratio of P507/Fe^3+^ of 1.5–1.7 was chosen as the optimum and was used for further optimization. Furthermore, scrubbing of the organic phase was done to remove impurities such as Cu, Mn, Ni, and Co. An attempt was made to scrub the organic phase with water only, as shown in Fig. 3b. The scrubbing efficiency for NMC metals was almost 100% at all O/A ratios. Moreover, water scrubbed 100% of Cu (around 0.051 g/L initially) from the loaded organic phase at an O/A phase ratio of 20, but the Li loss was very high, ~ 63% and was reduced to ~ 20% at an O/A ratio of 100. Despite this reduction, such a high phase ratio is not recommended from an industrial perspective due to potential entrainment losses, in addition to high Li loss. Li et al.^24^ suggested using a scrubbing solution of 0.5 mol/L HCl + 4.5 mol/L LiCl to scrub Mg^2+^ ions from a loaded organic phase of TBP and P507 from brine. They suggested that an ion exchange between Li^+^ from the scrub solution and impurity ions can purify the organic phase while preventing the loss of Fe and Li. Figure 3c shows the scrubbing of the organic phase with a scrub solution of 0.5 mol/L HCl + 3.5 mol/L LiCl. Even at an O/A ratio of 10, NMC and Cu scrubbing was 95% and 75%, respectively, with < 0.03% Fe loss while simultaneously loading 7–10% Li in the organic phase with respect to Li content initially loaded. The scrubbing efficiency of metals decreased with an increasing O/A ratio, which could be due to mass transfer limitations; therefore, an optimum O/A ratio of 10 was chosen. The scrubbed organic phase was further stripped with water at different O/A ratios, Fig. 3d. The Li stripping efficiency was 78% at an O/A ratio of 5, but Fe^3+^ loss was 7%. The Fe^3+^ loss decreased with increasing O/A ratio, such that it was < 1% at an O/A ratio of 20, but the same was true for Li, achieving 60% stripping at the same O/A ratio in a single stage. The lower % stripping of Li could be attributed to a lower P507/Fe^3+^ ratio, as a lower ratio hinders Li water stripping but encourages extraction and vice versa. The concentration of Li after single-stage stripping reached 13.5 ± 0.07 g/L from NMC 111 leachate and 11.3 ± 0.09 g/L from NMC 901 leachate, comparable to Su et al. for a similar system for brines^16^. The low stripping of Li made it necessary to either undergo multistage counter-current stripping or cross-current stripping. The multistage counter-current stripping of loaded organic phase is shown in Figs. 3e, and 4 counter-current stages were necessary at an O/A ratio of 13 to strip more than 85% of Li from the organic phase. The leftover Li ions must be removed by water washing or stripping at an O/A ratio of 20 to regenerate the solvent. Figure 3f presents the second option of cross-current stripping at an O/A ratio of 20, achieving concentrations of 13.5 g/L and 6.7 g/L from the first and second stage, respectively. This method resulted in around 99% Li stripping efficiency, effectively regenerating the solvent for the next extraction cycle. The two aqueous stripping streams can be combined before the recovery of Li salt.

#### Cyclic performance of solvents based on process scheme 1

The regenerated organic phase was used for the next cycle at an O/A ratio of 7 to extract Li from NMC 901 leachate. A higher O/A ratio was necessary to adjust the Fe^3+^/Li^+^ molar ratio of 1.3–1.7 and to compensate for P507 suppressing Li extraction. There was no need to spike the BM leachate with FeCl_3_ as the organic phase was already loaded with Fe^3+^. It must be mentioned that the NMC 901 leachate had around 3.7 g/L Fe^3+^ as a result of leaching the BM with HCl, Table 1. This was used as an advantage to make up for the minimal Fe^3+^ loss from the organic phase during stripping and regeneration stages. Figure 4 shows the cyclic performance of the solvents, where 1st cycle is done with TBP alone (O/A 4) with Fe-spiked NMC 111 leachate, Fig. 2, and all the remaining cycles (R1-R5) are done using NMC 901 BM leachate at an O/A ratio of 7. The Li extraction in a single stage in 1st cycle was 78% and was reduced to 63–65% in all the remaining cycles. Similarly, Fe^3+^ extraction was > 99% in cycles R1-R5, which provides two major advantages: (1) makes up for < 1% Fe^3+^ loss during stripping and regeneration stages, (2) essentially removes Fe^3+^ from the leachate, which is considered an impurity. The cycles R1-R5 indicated that as the equilibrium experimental studies were used in optimizing the parameters, the overall process was stable over multiple cycles. However, Li extraction efficiency was still lower, which could be due to competition between Li^+^ and H^+^ (free acid) in the BM leachates. This will be discussed in the subsequent sections.

The composition of strip solutions after various cycles is shown in Table 2. There was no detectable (ND) Ni, Co, or Mn with a few exceptions. The concentration of Li dropped from 13.5 g/L in the 1st cycle to around 11.3 g/L, after which it was stable. This initial drop may be attributed to differences in the leachate feed: NMC 111 was used in the first cycle, while NMC 901 was used in all subsequent cycles. The choice of different leachates was to show the application and performance of optimized conditions on handling different compositions of BM leachates. Additionally, the first cycle utilized only TBP, which likely resulted in a higher extraction of Li. The concentration of Fe^3+^ in the strip solution increased after subsequent cycles because, as mentioned previously, NMC 901 leachate had 3.7 g/L of Fe^3+^ after leaching. This Fe^3+^ was > 99% extracted in R1-R5 cycles, and it is possible that the solvent releases excess Fe^3+^ from the organic phase during water stripping.

**Table 2:** Composition of strip solutions after repeated cycles based on Fig. 4

Cycle	Fe g/L	Cu g/L	Ni g/L	Co g/L	Li g/L	Mn g/L
1st	0.7 ± 0.01	0.2 ± 0.01	ND	ND	13.5 ± 0.07	0.05 ± 0.0
R1	1.09 ± 0.0	0.2 ± 0.0	ND	ND	11.3 ± 0.09	ND
R2	1.2 ± 0.03	0.1 ± 0.0	ND	ND	11.3 ± 0.05	ND
R3	1.4 ± 0.02	0.2 ± 0.0	ND	0.01 ± 0.0	10.7 ± 0.1	ND
R4	1.5 ± 0.01	0.2 ± 0.0	ND	0.01 ± 0.0	10.7 ± 0.08	ND

Finally, it must be mentioned that some precipitation was observed in the raffinate after 1st cycle (with TBP alone) in some BM leachates over time, which could be due to TBP extracting HCl and water, and disturbing the solubility of other ions. Moreover, a volume reduction of the aqueous phase (raffinate) after extraction could be observed, further supporting this claim, which has also been reported in the literature^10^. To avoid this, Process Scheme 2 was developed.

### Process scheme 2

**Fig. 5:**
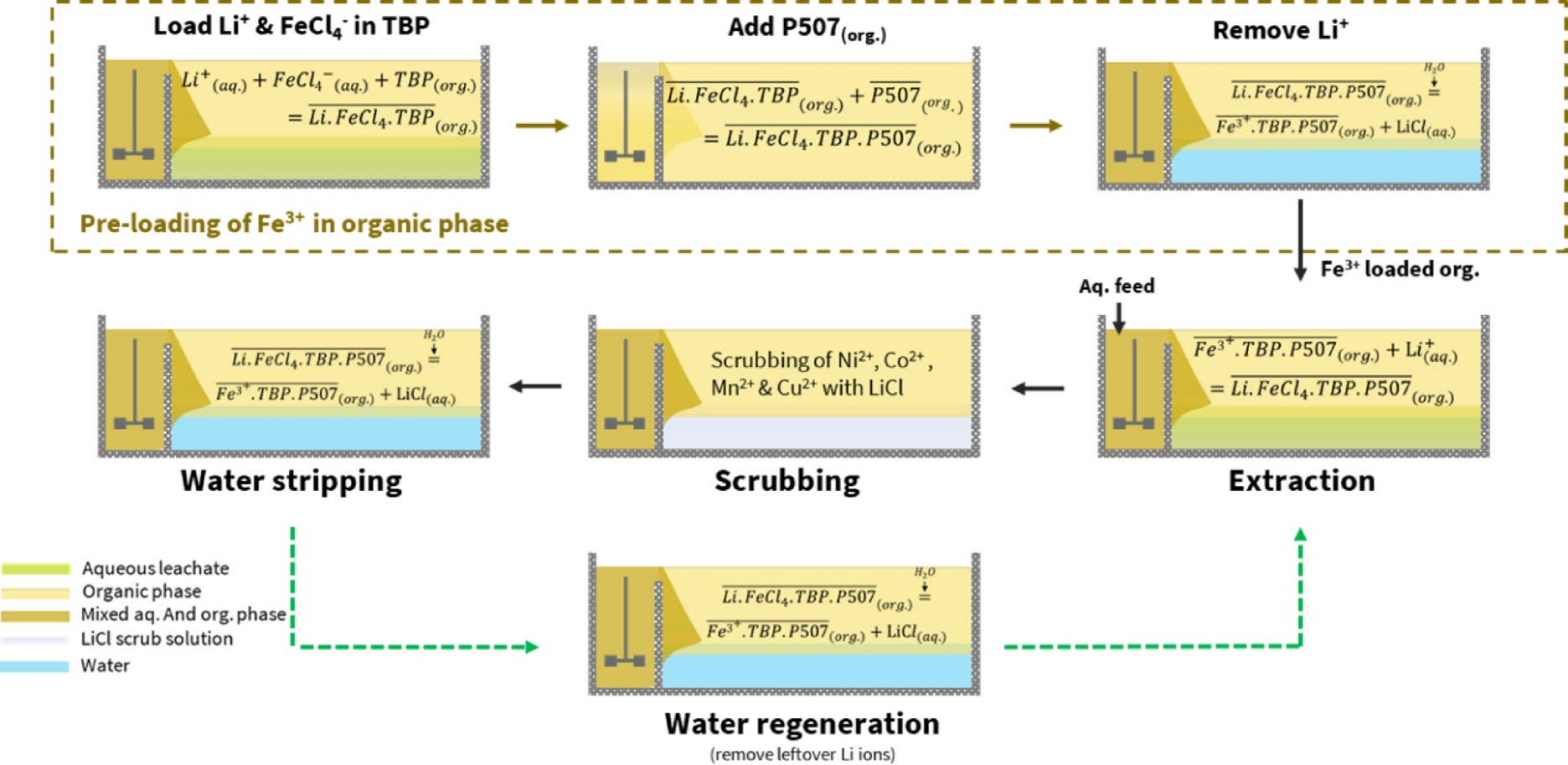
Process scheme 2.

Process Scheme 2, Fig. 5, was developed to separate the Fe^3+^ loading in the organic phase from a stock solution and Li extraction from the BM leachates. This was done to avoid precipitation in the raffinate as the BM leachates had ~ 1 mol/L of Li, which meant a Fe^3+^ concentration of 1.3 mol/L to adjust the Fe^3+^/Li^+^ molar ratio of 1.3. Such a high concentration gradient during extraction with TBP disrupts the aqueous phase solubility of other ions and contributes towards precipitation. That’s why a stock solution, in 0.5 mol/L HCl, of 2 g/L Li^+^ and 20 g/L Fe^3+^ was prepared while NiCl_2_ was added to adjust the Cl^−^ ion concentration to > 6 mol/L. The extraction was done with TBP only in an O/A phase ratio of 1, and after phase separation with centrifugation, P507 was added in the organic phase at a P507/Fe molar ratio of 1.5–1.7. Finally, Li was removed from the loaded organic phase with water stripping at an O/A phase ratio of 20, twice. The prepared organic phase had Fe^3+^ preloaded in nTBP.mP507 and was used to extract Li from the BM leachates.

#### Optimization of extraction stage and recyclability of organic phase

**Fig. 6:**
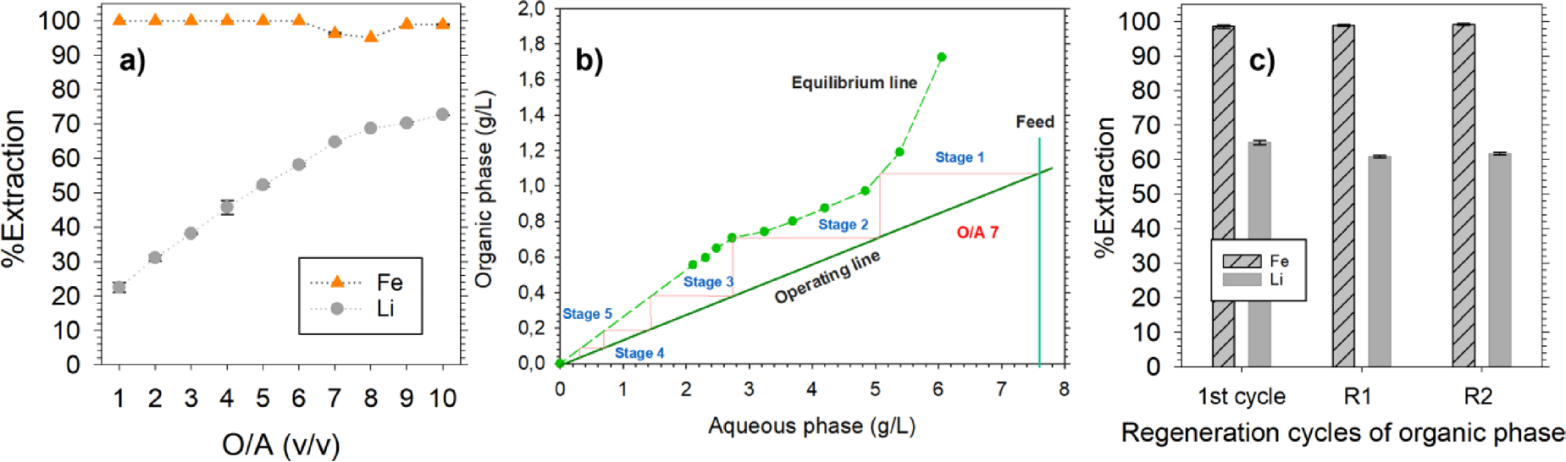
Optimization based on process scheme 2 where Fe^3+^ is preloaded in TBP and P507 (P507/Fe^3+^ molar ratio 1.5–1.7), (**a**) % Extraction vs O/A phase ratio from 1 mol/L NiCl_2_ spiked 901 leachate (**b**) McCabe–Thiele diagram for Li extraction based on 6a, (**c**) Regeneration cycles of organic phase based on process scheme 2 from 1 mol/L NiCl_2_ spiked 901 leachate.

Figure 6 illustrates optimization based on Process scheme 2. Only the extraction stage O/A phase ratio needed optimization as the organic phase had Fe^3+^ preloaded in both the solvents, unlike process scheme 1. The scrubbing, water stripping, and water regeneration conditions were the same as Process scheme 1, as they were optimized based on the equilibrium concentration of Li^+^ and Fe^3+^ in the loaded organic phase. Figure 6a shows Li and Fe extraction vs O/A phase ratio from NMC 901 leachate. A notable increase in Li extraction could be observed until an O/A phase ratio of 7, after which the increase becomes slow. The Li extraction efficiency at O/A phase ratio 7 was 65% which was similar to R1-R5 cycles in process scheme 1, Fig. 4. Similar extraction efficiency was obtained because Fe^3+^ concentration, during preloading, in the organic phase was adjusted in a way that at O/A 7 Fe^3+^/Li^+^ molar ratio becomes 1.3–1.7. This allowed for keeping the rest of the process, i.e., scrubbing, water stripping, and water regeneration, similar to process scheme 1, as the equilibrium concentrations of Fe^3+^ and Li^+^ in the loaded organic phase were similar in both. Figure 6b presents the McCabe–Thiele diagram for the extraction stage for process scheme 2. Four counter-current stages were necessary to extract > 92% Li from NMC 901 leachate containing ~ 8 g/L. It is important to note that the O/A phase ratio is also influenced by the concentration of Li^+^ in the feed solution. A lower operating ratio may be selected for solutions with lower initial Li concentrations. For instance, an O/A ratio of 1 was used when the Li concentration was 0.35 g/L, while an O/A ratio of 4 was employed at a concentration of 5.02 g/L in a simulated brine^15,16^. Even though a lower S/L ratio can be employed during leaching, resulting in a lower concentration of Li, it is preferred to have a higher concentration of metals in the leachate for easy processing while consuming the acid as well, in accordance with the principles of circular hydrometallurgy^25^. The loaded organic phase was scrubbed with 0.5 mol/L HCl + 3.5 mol/L LiCl solution at O/A phase ratio 10, stripped with water at O/A phase ratio 20, and regenerated with water at O/A phase ratio 20, similar to process scheme 1. The whole process was cycled three times, Fig. 6c. The extraction efficiency was maintained at 63–67% for all the cycles in a single stage, consistent with process scheme 1, Fig. 4. This confirmed that Fe^3+^ was stable in the organic phase and that the developed process can be recycled over multiple cycles. Even though the long-term stability of Fe^3+^ in the organic phase still needs further investigation, such a system (TBP and FeCl_3_) has already been applied on an industrial scale to produce Li from brines, with a capacity of 10,000 tons per year^26^. The solvent loss of a similar extraction system was reported to be around 150 mg/L over repeated cycles^11^. This demonstrates its potential for large-scale Li recovery from recycling streams as well. The two process schemes are also summarized in Table 3.

**Table 3:**
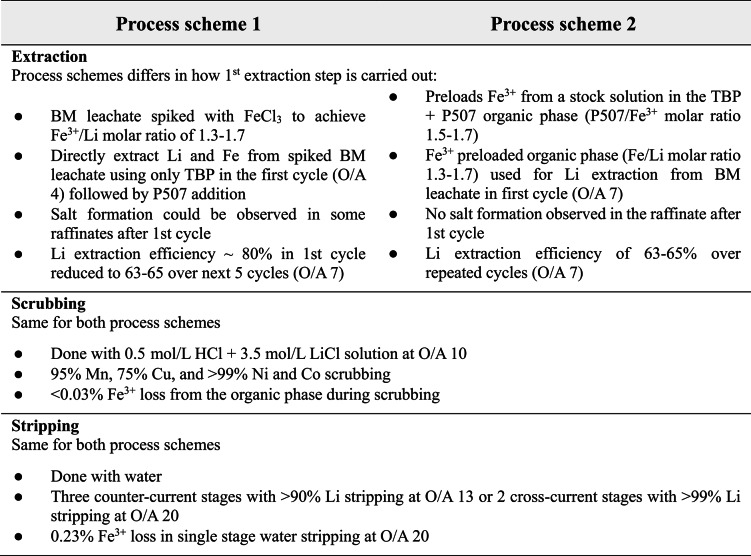
Summary and comparison of investigated process schemes.

### Effect of free acid on Li extraction

**Fig. 7:**
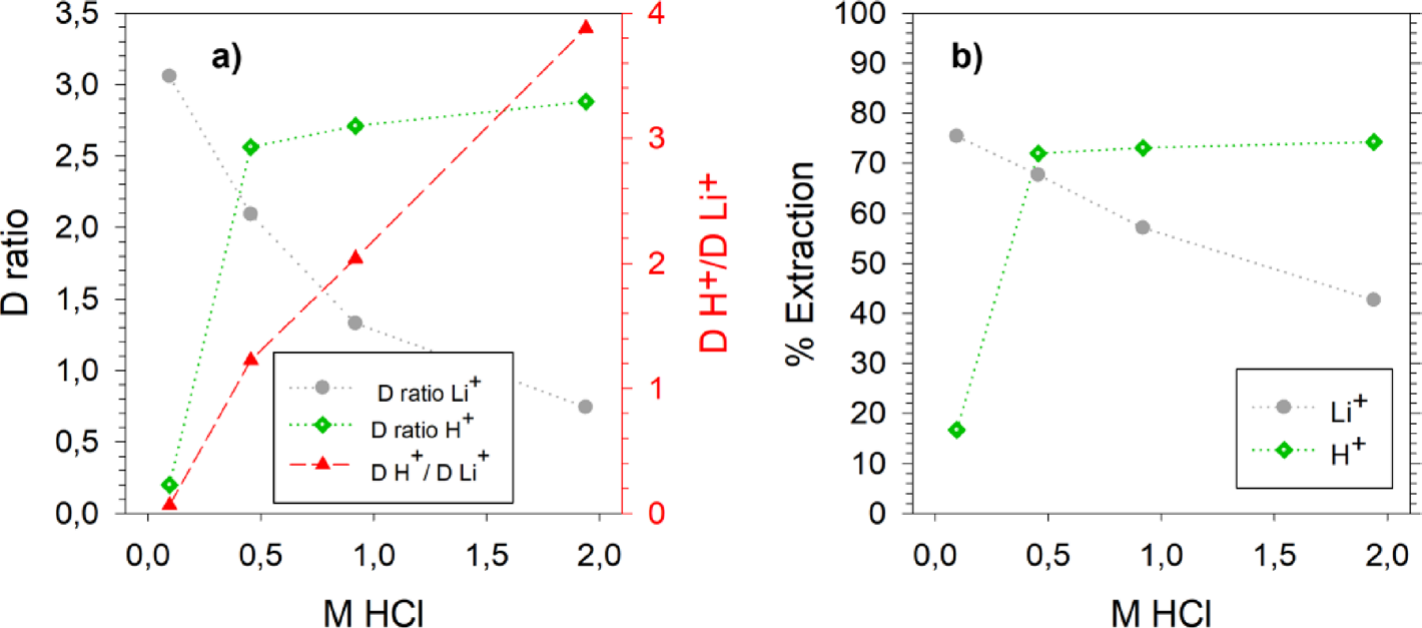
(**a**) Distribution (D) ratio of H^+^, Li^+^ vs concentration of HCl (mol/L), (**b**) H^+^ and Li^+^ extraction from stock leachates containing varying amount of HCl.

As mentioned earlier, the Li extraction efficiency from the BM leachates was low, ~ 65% in a single stage, and could be improved significantly. This was suspected due to the presence of free acid in the BM leachates, which may compete with Li during extraction. Table 1 presents the composition of the three BM leachates, which were leached with 6 mol/L HCl. The free acid concentration in all the leachates was above 0.7 mol/L, with NMC 901 having the highest concentration of 0.92 mol/L. To investigate the potential competition between H^+^ and Li^+^ ions, stock solutions were made with a Li^+^ concentration of 1.01–1.09 mol/L, Fe^3+^  ~ 0.05 mol/L, 2.6 mol/L NiCl_2_, and varying concentrations of HCl ranging from 0.1 to 2 mol/L. Figure 7a illustrates the distribution (D) ratios of H^+^ and Li^+^ versus different concentrations of free acid in the stock leachate. A sharp rise in the D ratio of H^+^ can be seen from 0.1 to 0.5 mol/L HCl. Similarly, the D ratio of Li^+^ realizes a sharp decline from > 3 at 0.1 mol/L HCl to ~ 0.7 at 2 mol/L HCl. This decrease in Li^+^ D ratio with increasing HCl concentration indicates that H^+^ competed with Li during extraction. The ratio of D H^+^ and D Li^+^ further shows an almost linear increase with increasing HCl concentration, which supports this hypothesis. The Li extraction efficiency from the stock solution containing 7.6 g/L Li and 0.1 mol/L HCl was ~ 76% which was reduced to ~ 57% from a similar stock solution containing 1 mol/L HCl, Fig. 7b. The free acid concentration in NMC 901 leachate, Table 1, was 0.92 mol/L and gave Li extraction of 63–67% which is comparable to afore mentioned Li extraction from the stock leachates. Su et al.^16^ reported a decrease in Li extraction from 84.5 to 67.4% when HCl concentration was increased from 0 to 0.25 mol/L using Fe^3+^ preloaded TBP and P507 from brine. They suggested maintaining the HCl concentration as low as possible because H^+^ ions can be extracted as H.FeCl_4_.nTBP over Li^+^ ions^16^. Finally, to increase Li extraction from the BM leachates, the free acid could be reduced by either choosing a higher S/L ratio or a lower concentration of acid during leaching, extracting the excess acid or neutralizing it up to 0.1 mol/L.

## Mechanism of extraction

**Fig. 8:**
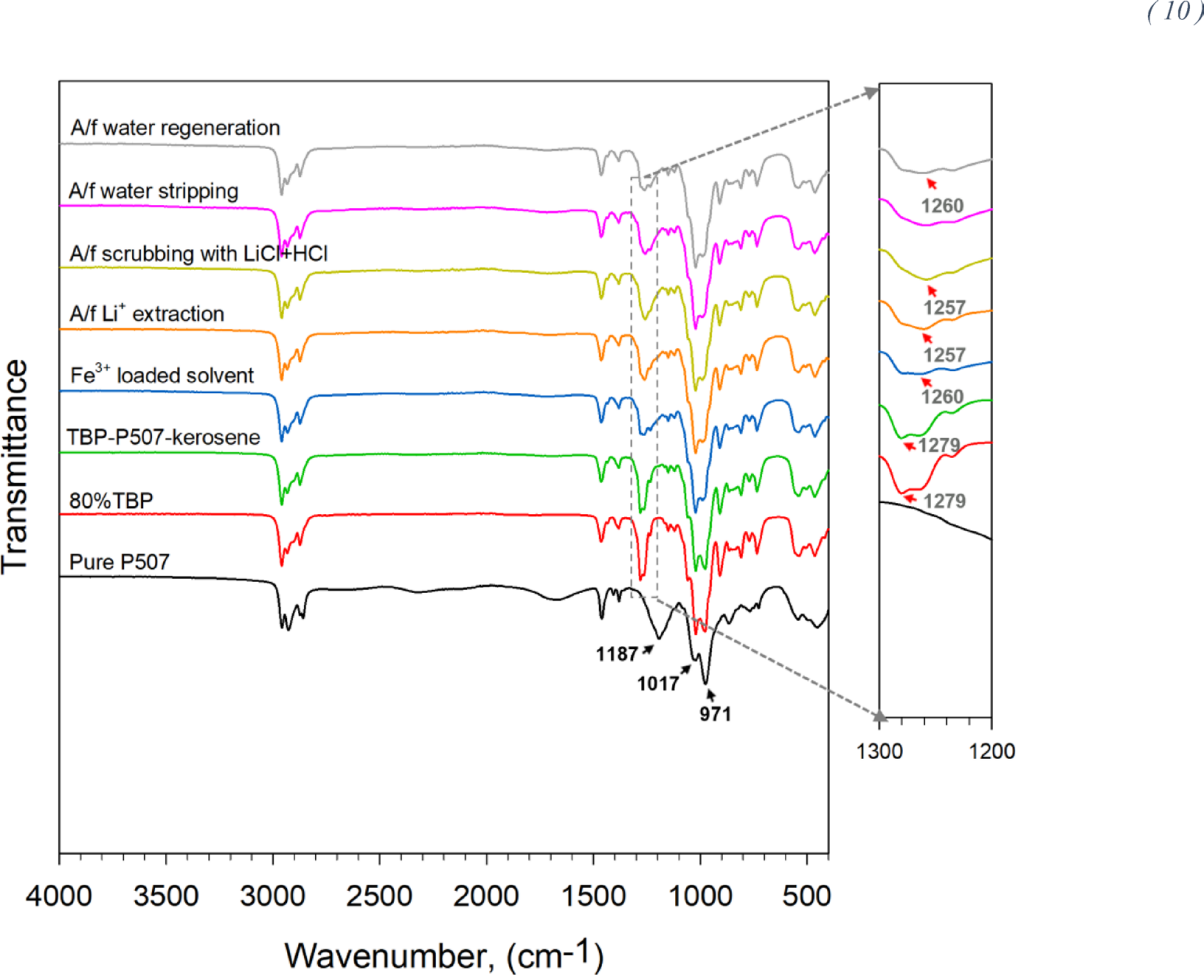
FTIR spectra of pure and loaded organic phase.

The FTIR spectra of the organic phase after different process stages is shown in Fig. 8. The pure P507 displayed distinct bands at 1187, 1017, and 971 cm^−1^ attributed to stretching vibration of P=O, P–O–C, and P–O–H^27^, respectively. Moreover, 80% TBP in kerosene shows a distinct peak at 1279 cm^-1^ attributed to the stretching vibration of P=O, which plays a key role in coordinating with Fe^3+^ and other metal ions^28^. The mixed organic phase of TBP, P507, and kerosene retains the 1279 cm^−1^ band attributed to TBP. Notably, this band underwent a significant shift to 1260 cm^−1^ when Fe^3+^ is preloaded in the organic phase (preloading procedure explained in process scheme 2, Fig. 5)^28^. The shape of the spectra further changes when Li is loaded in the Fe^3+^ preloaded solvent from BM leachate and remains after scrubbing of the organic phase with HCl + LiCl solution. More Li (7–10%) was loaded during scrubbing. After water regeneration, the peak at 1260 cm^−1^ became clearly visible again, confirming that Fe^3+^ was retained in the solvent. This observation further verifies that adding P507 as a cosolvent coordinates with Fe^3+^, along with TBP, stabilizing it in the organic phase under low Cl^−^ concentration during water stripping. Li et al.^24^ studied the application of a similar system for Li extraction over magnesium and suggested that the coordination mode of Fe changes with varying Cl^-^ concentration. At high Cl^-^ concentrations, Fe^3+^ predominantly exists as the FeCl_4_^−^ anion, which is extracted by TBP along with Li^+^. However, under low Cl⁻ concentrations, the coordination mode of Fe^3+^ becomes more complex, as represented in Eq. (10)^24^.

10 $${\mathrm{Li}}.{\mathrm{FeCl}}_{4} .{\mathrm{nTBP}}.{\mathrm{mP}}507_{{\left( {{\mathrm{org}}.} \right)}} \frac{{{\mathrm{H}}_{2} {\mathrm{O}}}}{ \Leftrightarrow } ({\mathrm{FeCl}}_{2} .{\mathrm{nTBP}}.{\mathrm{mP}}507){\mathrm{Cl}}_{{\left( {{\mathrm{org}}.} \right)}} + {\text{ LiCl}}_{{\left( {{\mathrm{aq}}.} \right)}}$$

**Fig. 9:**
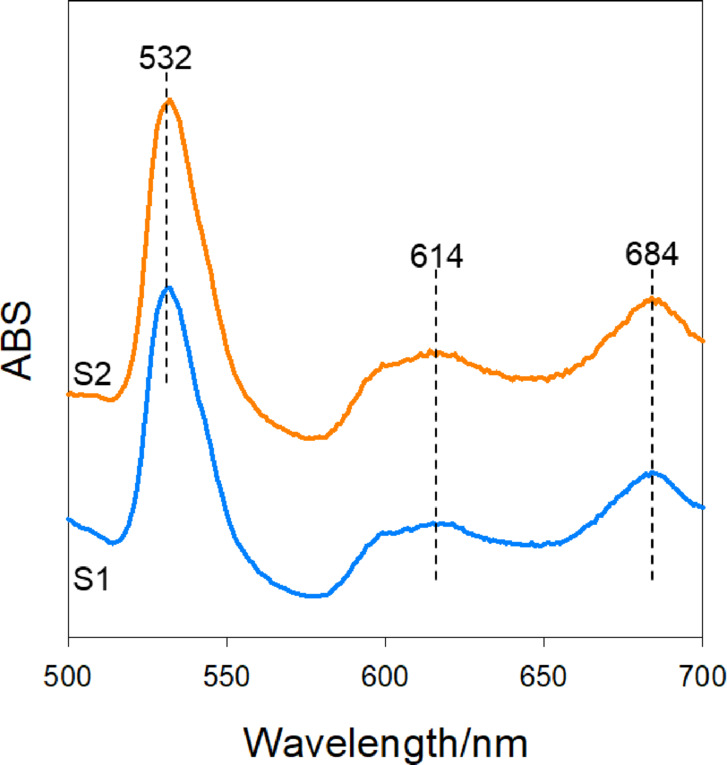
UV–Visible spectra of organic phase, S1—Fe^3+^ preloaded in TBP and P507 before Li extraction, S2—after Li extraction from 1.01–1.09 mol/L Li^+^, 0.05 mol/L Fe^3+^, 2.6 mol/L NiCl_2_ and 0.1 mol/HCl leachate.

The UV–Vis spectra of the organic phase at different process steps are shown in Fig. 9. The peaks observed at 532, 614, and 684 nm correspond to characteristic absorption peaks of FeCl_4_^−^ confirming its speciation in the organic phase^15,17^. The presence of FeCl_4_^−^ in the organic phase at lower Cl^−^ concentration is also reported in the literature^17^. However, the intensity of spectrum before Li extraction (S1) is lower compared to after (S2). This further confirms that during Li extraction, high Cl^−^ concentration promotes the formation of FeCl_4_^−^, which is extracted in the organic phase along with Li^+^. While the UV–Vis and FTIR spectra confirm that Fe^3+^ is stabilized in the organic phase, thereby supporting the recyclability data, it is essential to understand the coordination behavior of Fe^3+^ in detail under low Cl^−^ concentrations. This understanding could help improve the efficiency of lithium water stripping and facilitate easier solvent regeneration.

### Early Li extraction and overall battery recycling process

In addition to eliminating the need for thermal pretreatment of BM, the solvent extraction of Li as the first metal ion has the potential to simplify the overall recycling process. After the recovery of Li, already reported separation routes can be adopted to purify the aqueous raffinate. First, leftover Al and Cu may be recovered as hydroxide at moderate pH (around 5), followed by oxidation of Mn^2+^ and Co^2+^, and finally Ni^2+^ precipitation as hydroxide^19^. After removal of leftover Fe^3+^ by hydrolysis, battery-grade products can be produced from Li rich strip solution. For example, Li_2_CO_3_ can be precipitated by adding sodium carbonate^22,29^, or electrodialysis/solvent extraction can be used to convert LiCl followed by antisolvent crystallization or carbonation to produce LiOH^30–32^.

The approach studied offers significant improvements over previous reports. Wesselborg et al.^10^ investigated the application of such a system for Li extraction from synthetic LIB waste leachate using only TBP, reporting excellent selectivity over Ni^2+^ and Co^2+^ ions. However, they preloaded the organic phase with NaFeCl_4_.nTBP and released Na^+^ in the raffinate during extraction. In addition, without the use of Fe^3+^ stabilizing co-solvent, they employed 6 mol/L HCl and obtained around 2 mol/L Li in the stripping solution. This resulted in a highly acidic strip solution and organic phase, which needed to be neutralized/saponified before the next cycle. Su et al.^16^ reported the application of a ternary solvent system (FeCl_3_, TBP and P507) from brines where MgCl_2_ was used as the chloride source and for preloading of Fe^3+^ in the organic phase. However, the chemistry of LIBs leachate is complex, where the introduction of impurity ions must be avoided due to the presence of high-value metals (Ni^2+^, Mn^2+^ and Co^2+^) in the raffinate. In this study, the use of acids, alkalis, and impurity introduction during extraction is avoided. Furthermore, the use of a high S/L ratio during leaching and NiCl_2_ as a chloride source serves a more sustainable approach as the market also shifts towards high nickel cathode chemistry. This method aligns with the principles of circular hydrometallurgy, which emphasize circular flowsheets, minimizing the use of reagents, preventing waste, maximizing mass and energy efficiency, and regeneration of reagents, among others ^25^. A comparison of the studied process with recent reported literature is summarized in Table 4.

**Table 4:** Summary and comparison of recent reported literature on Li solvent extraction.

Feed composition (g/L)	Organic phase and stripping reagents	Performance	Cycles	References
Simulated brine, 0.35 Li, 96 Mg, 1.84 Na and 0.62 K	TBP and FeCl_3_, stripping with HCl + NaCl, regeneration with MgCO_3_ and Mg(OH)_2_	65% single-stage Li extraction, Li/Mg 347, Li/Na 29.1, Li/K 202.02	10 with 53% Li extraction	^12^
Simulated brine, 0.279 Li and 110.7 Mg	TBP and FeCl_3_, stripping with 6 M HCl needing NaOH for saponification	67.9% single-stage Li extraction, Li/Mg = 435.5 with 28.8 g/L Li and 0.6 g/L Mg in strip solution	20	^11^
Simulated brine, 1.99 Li and 97.2 Mg	TBP, FeCl_3_ and Cyanex 272, stripping with water	81% Li extraction	6 with extraction maintained	^13^
Simulated brine, 0.35 Li, 97.2 Mg, 1.84 Na and 0.78 K	TBP, FeCl_3_ and P204, stripping with water	52.71% single-stage Li extraction	NA	^15^
Simulated brine, 0.69 Li and 97.2 Mg	TBP, FeCl_3_ and P507, stripping with water	57% single-stage Li extraction, 8.25 g/L Li and 9.16 g/L Mg in strip solution	NA	^17^
Salt lake brine, 5.02 Li, 108.1 Mg, 1.31 Na, 0.59 K and 2.23 B	TBP, FeCl_3_ and P507, stripping with water	20.92 g/L Li, 2.23 g/L Mg and 1.61 B in strip solution	NA	^16^
Synthetic BM leachate, 2.5 Li, 1.4 Al, 15 Co, 2.1 Cu, 2 Mn and 1.9 Ni	TBP and FeCl_3_, stripping with 6 M HCl needing NaOH for saponification	87% Li extraction, Li/Ni = 2825, Li/Co = 854 with 13.95 g/L Li in strip solution	NA	^10^
Industrial BM leachates, 8–9 Li, 25–64 Ni, 8–14 Co, and 0–20 Mn	TBP, FeCl_3_ and P507, stripping with water	65% single-stage Li extraction increased to 76% with controlled acidity, 11–14 g/L Li and up to 0.05 g/L Mn in strip solution	6 with extraction maintained	This study

## Conclusion

In this study, selective extraction of Li from acidic chloride leachates of spent batteries black mass (BM) has been investigated. A ternary organic phase consisting of Fe^3+^, TBP, and P507 to selectively extract Li^+^ over Ni^2+^, Mn^2+^, and Co^2+^ has been developed. The extraction, scrubbing, stripping, and regeneration conditions were optimized, and McCabe–Thiele diagrams were constructed. The P507/Fe^3+^ (optimum 1.5–1.7) mole ratio was found to be a critical parameter, as an increasing ratio enhanced Li release during water stripping but suppressed extraction. The extraction mechanism was studied with FT-IR spectroscopy, which confirmed the stabilization of Fe^3+^ in the organic phase under low Cl^−^ ion concentration. Two process schemes were developed, which differed from each other in performing the first extraction. Process scheme 1 directly loaded LiFeCl_4_ in TBP, achieving ~ 80% Li extraction at organic-to-aqueous (O/A) ratio 4 in the first cycle, reduced to ~ 65% (O/A 7) in the subsequent cycles due to the addition of P507. TBP extracts HCl and H_2_O, resulting in precipitation in some leachates during 1st cycle, leading to the development of process scheme 2. In this second process Fe^3+^ was preloaded in the organic phase from a stock solution and giving > 90% Li (~ 8 g/L initially) extraction at O/A 7 in four stages with a single-stage efficiency of ~ 65%. The lower Li extraction was due to the presence of free acid in the leachate, which competed with Li as extraction increased to 76% from a stock leachate containing 7.6 g/L Li^+^ and 0.1 mol/L HCl at O/A 7. After six cycles, the Li extraction efficiency remained at ~ 65% in single-stage, showing excellent selectivity, with BM feed concentrations (g/L) of 8–9 Li, 25–64 Ni, 8–14 Co, and 0–20 Mn, and strip solution (g/L) of 11–14 Li and 0–0.05 Mn, with no detectable Ni and Co in single-stage water stripping. The developed process can be easily integrated into conventional battery recycling flowsheets, where Li could be the first element to be recovered from a total leachate. The process negates the need for thermal pretreatment of BM and does not release any impurities during extraction, while also eliminating the need for acids and bases.

## Data Availability

The datasets generated during and/or analysed during the current study are available from the corresponding author on reasonable request.
